# Pituitary Extract

**Published:** 1919-11-29

**Authors:** 


### PITUITARY EXTRACT.

### Some Applications and their Value.

Pituitary extract should certainly be placed among the most valuable drugs in the British Pharmacopœia, and its uses are so manifold that every up-to-date medical man, nurse, or midwife should have a working knowledge of its actions, properties, indications, mode of administration, and correct dosage. In order fully to appreciate the actions of this drug, and to understand this subject properly, one must first realise, in outline at least, the anatomical and physiological characters of the gland.

### Anatomy of the Gland.

The pituitary body (hypophysis) is found at the base of the brain and occupies the "sella turica," or upper hollowed-out surface of the sphenoid bone. The gland is flattened from above downwards, is ovoid in shape, and has its long axis directed transversely. For the purposes of description the gland is divided into two lobes, an anterior and a posterior. Histologically the anterior lobe consists chiefly of secreting epithelium not unlike that of the thyroid, whilst the posterior portion is composed of fibrous tissue containing blood-vessels and a few branched cells scattered throughout it. Developmentally thereto it is interesting to relate the entirely different origin of the two lobes, and this fact will enable us the more easily to understand their different physiological properties.

The anterior lobe is developed from a tubular diverticulum of the buccal epithelium, whilst the posterior originates as a tubular downgrowth of the cerebrum or brain proper in the region of the floor of the third ventricle.

### Physiology.

The anterior lobe is generally regarded as the functional portion of the gland, and in some way regulates the growth of the body; undue size of this portion of the gland gives rise to giantism and the disease known as acromegaly, whilst the experiment of ablation of this portion has always resulted in death. Extracts of the anterior portion have no special effect on the circulation other than the slightly depressor effect commonly produced by tissue extracts.

On the contrary, aqueous extracts of the posterior portion have the most profound effect on the body organism, and it is with these effects that we are concerned in this article. The aqueous extract most commonly known to the medical profession is pituitrin, which is the trade name under which it is sold by one firm; other firms have other names, but pituitary extract covers them all; it is preserved with chloretone, and one cubic centimetre represents 0.2 grm. of the fresh substance.

It is of the utmost practical importance to remember that this solution, in common with other solutions of animal origin, is liable to deteriorate with age. As a rough working rule no solution should be kept for more than about five months; however, most manufacturers print these details on the labels of bottles containing this extract. Another thing to remember is to keep the bottle well corked, and to keep it in a dark place away from the sunlight, else again the solution will rapidly lose its efficiency. Sealed ampoules get over much of this drawback.

### Dosage and Actions of the Drug.

The drug is usually administered as an intramuscular hypodermic injection in a dose of from 0.5 to 1 cc. (8½ to 17 minims), or orally in doses ranging from 10 to 30 minims. Pituitary may also be given as an intravenous injection in doses of 0.12 to 1 cc. (2 to 17 minims), diluted with warm physiological sodium chloride solution. This extract is a stimulant of the arterial system, and has a selective activity on the unstriped involuntary musculature of the pregnant uterus, the intestine and the bladder.

### The Effect Upon the Blood Pressure.

Normal blood pressure is maintained partly by the heart's action and partly by the vaso-motor system. The arterioles are invested by unstriped muscles, and contraction or relaxation of these will lower or heighten the blood pressure: the rate of the heart-beat being quickened when the pressure is low, and *vice versâ*. Extracts of pituitary will cause a constriction of the end arterioles with a corresponding rise in blood pressure and a corresponding relief of a rapidly beating heart.

Unlike adrenalin, repeated doses of pituitary have no beneficial effect—on the contrary, a depressor effect is attained; thus an interval of at least four hours should exist between consecutive doses.

### A Valuable Drug in Midwifery.

Strong emphasis should be laid on the fact that the drug is only active upon a uterus which is actually in labour, otherwise it would be a most valuable abortifacient. The two obstetric conditions best affected by pituitary are delayed labour and post-partem hæmorrhage: but judicious knowledge and some experience are requisite for an optimum effect of the drug and its safety, so far as the patient is concerned. First, in regard to delayed labour: when this is solely due to uterine inertia the drug initiates powerful contractions and some retraction of the upper or propensile segment of the uterus; the lower uterine segment then dilates, and labour continues with accelerated speed. This treatment is admirable in skilled hands, but has very many pitfalls for the unwary. Thus if the delayed labour is due to an obstruction caused either by the posture of the fœtus *in utero* or to a pelvic abnormality, the uterus contracts violently, and rupture may result. The golden rule is never to administer the drug unless the position of the child be normal, and the maternal passages quite clear of any obstruction. The question of a suitable dosage is again one only to

**Pituitary Extract**—(*continued*).

be arrived at after long experience; suffice it to say that the beginner should administer only medium doses, say, half a cubic centimetre, lest precipitate labour result. The risks of too precipitate labour are very definite; they include cervical laceration and possible rupture of the posterior vaginal wall and perineum. Either of these accidents is a calamity to the mother too serious to be regarded with complacence.

The initial experiments for the determination of the pre-parturient effects and dosage of this drug were chiefly carried out on actual mothers in Germany where, with a ruthless disregard of human life, these accidents were often witnessed. Indeed, several cases of acute obstetric inversion of the uterus were recorded amongst these experiments.

### Use in Post-Partem Hæmorrhage.

Here again caution is needed, for there are several causes of post-partem hæmorrhage, such as a ruptured cervix, posterior vaginal wall, or perineum, upon which pituitary has little or no effect.

The specific action of pituitary extract in this condition depends upon its selective activity upon the uterine muscle: blood is escaping from the placental site. and is stopped by the contraction and retraction of the musculature upon the blood-vessels which course through it.

### A Contra-indication.

The drug should never be administered to a subject exsanguine from a great loss of blood, such as a post-partem hæmorrhage, as this may result in a sudden collapse, due to cerebral anæmia, which may often terminate in death itself. The exact pathology of this collapse is not fully understood; numerous theories, more or less convincing, have been advanced, but as a clinical fact it is generally recognised. More than one death was observed in one of our great London hospitals from this cause in the early days of the use of pituitary in obstetric practice.

### Use in Ileus and Acute Post-Operative Dilatation of the Stomach.

Post-operative distension of the bowel is a distressing condition for both patient and medical man, and is due to a temporary loss of contractile power in the gut wall. Now pituitary will stimulate the muscle to contract, and thus tend to cure the condition. The dosage under these circumstances need not be a maximum, as clinical experience has convinced us that injections of three to four minims have usually sufficed for this purpose. Inasmuch as a certain amount of time has to elapse between successive doses of pituitary, eserine sulphate (one-sixtieth of a grain) is an excellent drug to give in the interval, should the patient's general condition make this course advisable.

